# Leiomyosarcoma of the Great Saphenous Vein: A Case Report and Review of the Literature

**DOI:** 10.1155/S1357714X04000234

**Published:** 2004-12

**Authors:** Alexander G. J. Marle, Maarten W. G. A. Bronkhorst, Mark A. M. Brouwers

**Affiliations:** Department of Surgery Leyenburg Hospital Leyweg 275 The Hague 2545 CH The Netherlands

## Abstract

*Introduction:* Leiomyosarcoma of the venous system is rare, even more so in the greater saphenous vein. In the 85 years since van Ree described the first case in 1919 only 25 cases have been reported in the world.

*Methods:* We describe a case of an 85-year-old woman who was successfully treated by excision of the tumour. We also reviewed pertinent literature with regard to age, gender spread, tumour size, survival, occurrence of metastases and therapy.

*Results:* The median age was 54 years (range 2–85 years), with a 3:2 female to male ratio. The median size of the tumours was 4.1 cm (range 2–12 cm) and metastases occurred in seven of the 25 cases. If any form of adjuvant therapy is used it is usually radiotherapy. Chemotherapy seems to be reserved for cases where metastasis occurs. Average survival was 4 years (range 1 month to 17 years). Currently the best treatment seems to be wide excision of the tumour, with selective vascular reconstruction combined with adjuvant radiotherapy.


*Sarcoma*, December 2004, *VOL. 8*, *NO. 4*, *135–139*

Taylor & Francis healthsciences

ORIGINAL ARTICLE

### Leiomyosarcoma of the great saphenous vein: a case report and review of the literature

ALEXANDER G.J. VAN MARLE, MAARTEN W.G.A. BRONKHORST & MARK A.M. BROUWERS

*Department of Surgery, Leyenburg Hospital, Leyweg 275, 2545 CH The Hague, The Netherlands*

**Abstract**

*Introduction:* Leiomyosarcoma of the venous system is rare, even more so in the greater saphenous vein. In the 85 years since van Ree described the first case in 1919 only 25 cases have been reported in the world.
*Methods:* We describe a case of an 85-year-old woman who was successfully treated by excision of the tumour. We also reviewed pertinent literature with regard to age, gender spread, tumour size, survival, occurrence of metastases and therapy.
*Results:* The median age was 54 years (range 2–85 years), with a 3:2 female to male ratio. The median size of the tumours was 4.1 cm (range 2–12 cm) and metastases occurred in seven of the 25 cases. If any form of adjuvant therapy is used it is usually radiotherapy. Chemotherapy seems to be reserved for cases where metastasis occurs. Average survival was 4 years (range 1 month to 17 years). Currently the best treatment seems to be wide excision of the tumour, with selective vascular reconstruction combined with adjuvant radiotherapy.

**Key words:** *leiomyosarcoma, great saphenous vein*

### Introduction

Primary malignant venous tumours are very rare. Leiomyosarcoma of the saphenous vein even more so, constituting about one in every million malignant tumours. In the 85 years since Van Ree reported the first case in 1919, only 24 cases have been reported in the international literature. We describe an additional one, and pertinent literature is reviewed.

### Case

An 85-year-old woman was admitted to our hospital with loss of weight and general discomfort. She also complained of a progressive and painful swelling in the right groin, which she had noticed 3 weeks before. She had no relevant medical history.

On physical examination the woman was seen to be cachectic. A solid swelling with a diameter of 3 cm was observed in the right groin inferior to the inguinal ligament. The swelling was mobile in relation to the skin but fixed to a deeper structure. It was thought to be an enlarged lymph node. Further physical examination showed no other abnormalities.

Additional ultrasound and MRI of the right groin showed a tumour 2.5 cm in diameter in close proximity of the femoral vein (without obstructing it) and the cortex of the pubic bone just below the skin. Ultrasound guided biopsy showed leiomyosarcoma grade II. Preoperative workup showed no evidence of metastases.

During surgery we observed that the tumour originated from the great saphenous vein close to its insertion in the common femoral vein (Fig. 1) The tumour was excised with a wide margin.

Pathological examination of the excised tumour showed a leiomyosarcoma grade II with a diameter of 3.6 cm radically excised and four lympnodes with reactive changes but no signs of metastasis.

Postoperative course was complicated by a wound infection, which was contained with a wound debridement and antibiotics. There were no further complications and the patient made a slow but full recovery. After 27 days she was discharged from the hospital.

Correspondence to: Alexander Geert Jaap van Marle, Department of Surgery, Leyenburg Hospital, Leyweg 257, 2545 CH The Hague, The Netherlands. Tel.: +31-65-2460084; Fax: +31-70-3592477; E-mail: agjvanmarle@hotmail.com

1357-714X print/1369–1643 online/04/040135–5 © 2004 Taylor & Francis Ltd
DOI: 10.1080/13577140400023610

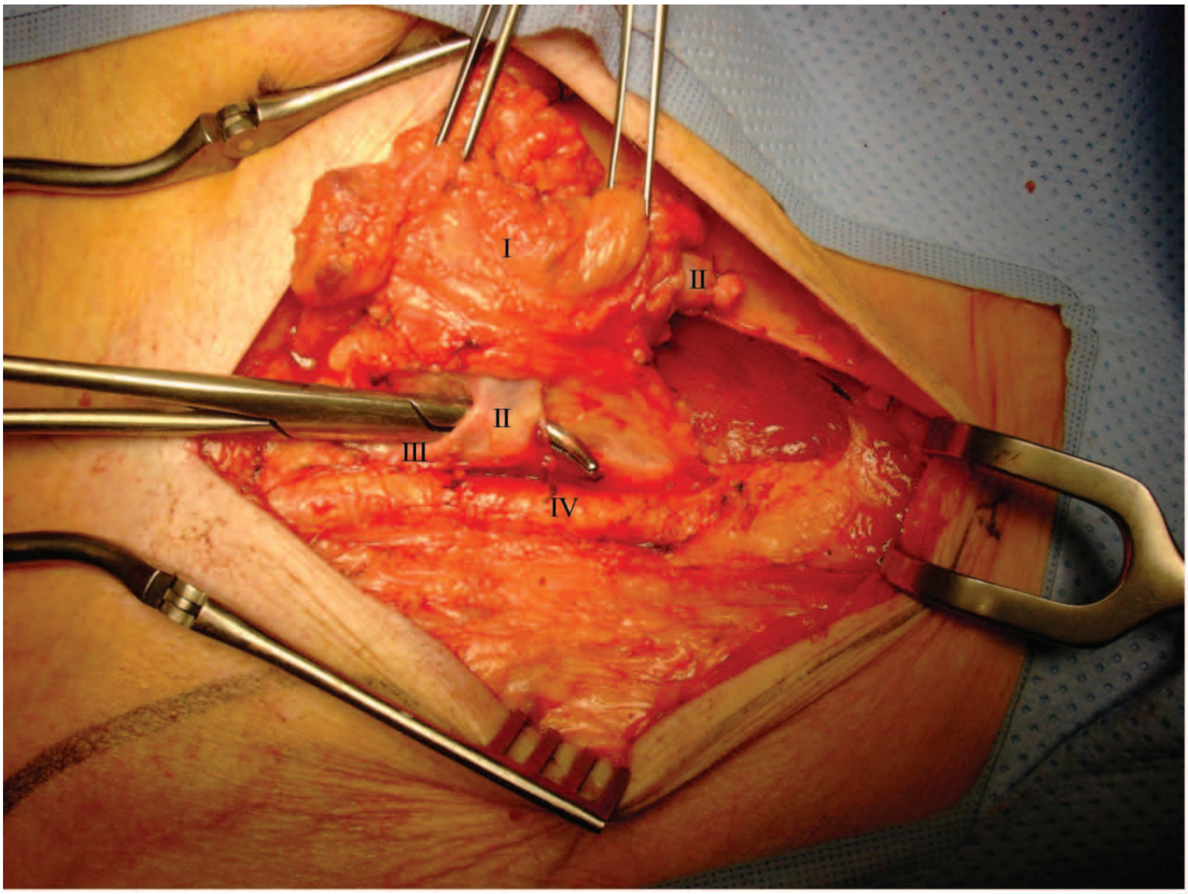

**Fig. 1.** *Leiomyosarcoma of the greater saphenous vein. (I) Leiomyosarcoma; (II) greater saphenous vein; (III) common femoral vein; (IV) common femoral artery. The head of the patient is on the left hand side.*

She refused adjuvant radiotherapy. At her last outpatient visit, 2 months after the surgery, the wound had fully healed, and she suffered no effects from surgery.

### Discussion

Malignant tumours that arise from venous walls are very uncommon, even more so in the lower extremity.[1,3] Primary venous leiomyosarcomas constitute less than one in every 100,000 malignant tumours, and about 10% of these originate from the great saphenous vein, with the inferior caval vein being the predominant location. This case is the second reported in the Dutch literature, and only the 25th in the international literature. The international literature consists mostly of case reports. There are only a few reviews and series.

Reix *et al.*[1] describe a series of seven patients with a venous tumour, six with a leimyosarcoma and one with hemangioendothelioma. Four of them originated from the superficial femoral vein, two from the common femoral vein and only one originated from the great saphenous vein. Median survival in this group was 31 months. Most of them died from metastasis. Local recurrence was never observed. Of the patients with leiomyosarcoma, four received adjuvant radiotherapy (60 Gy in all cases), one refused; and in one case was not indicated because the tumour was small and wide, and wide excision was performed. Chemotherapy was only offered in case of metastasis. Prognosis was poor due to early occurrence of metastasis, occurring in six of the seven cases.

In the same institute, a study was conducted to assess the best imaging technique for diagnosing venous tumours. It was concluded that echo duplex and MRI were best suited for diagnosis of venous tumours.[2] This was confirmed by Dzsinich *et al.*[3]

The latter author described a series of 13 patients with a primary venous leiomyosarcoma.[3] Of these 13, only two had a tumour arising from the greater saphenous vein. Most occurred in the inferior caval vein. At the time of their publication they state that only 197 cases of primary venous leiomyosarcoma had been reported in the period of over 100 years since Perl[4] reported the first in 1871. Of these, only 19 originated from the greater saphenous vein.

Humphrey *et al.*[5] described a case and reviewed the literature. At the time of their publication in 1987, only 15 cases of a leiomyosarcoma originating from the great saphenous vein had been reported,

**Table 1.** *Reported cases of leiomyosarcoma of the saphenous vein*

| Case | Author | Year reported | Age | Sex | Size (cm) | Metastasis | Local recurrence | Adjuvant RT/Chemo | Secondary RT/Chemo | Follow-up | Outcome |
|---|---|---|---|---|---|---|---|---|---|---|---|
| 1 | Van Ree | 1919 | 42 | F | * | No | No | No | No | 15 months | Alive, no evidence of disease |
| 2 | Smout | 1960 | 76 | F | 4 | No | No | Yes, 6000 Rad | No | 8 years | Death, no evidence of disease |
| 3 | Dorfman | 1963 | 56 | M | 3 | No | No | No | No | 1 year | Alive, no evidence of disease |
| 4 | Cristiansen | 1964 | 68 | F | 4.5 | No | No | No | No | 2 months | Alive, no evidence of disease |
| 5 | Allison | 1965 | 3,5 | F | 3.5 | No | No | No | No | 6 months | Alive, no evidence of disease |
| 6 | Leu | 1969 | 40 | M | * | No | No | No | No | 2.5 years | Alive, no evidence of disease |
| 7 | Szaz | 1969 | 68 | M | 5.5 | Liver, cheek | Yes | No | No | 4 years | Death, liver failure |
| 8 | Hughes | 1973 | 53 | F | 2.5 | No | No | No | No | 6 months | Alive, no evidence of disease |
| 9 | Jenstrom | 1975 | 64 | M | 12 | No | No | No | No | 14 months | Alive, no evidence of disease |
| 10 | Gross | 1975 | 46 | M | 5 | Thyroid, subcutaneous | No | No | Yes, RT 6500 Rad | 3 years | Alive, no evidence of disease |
| 11 | Stringer | 1977 | 39 | M | * | No | No | No | No | 8 years | Alive, no evidence of disease |
| 12 | Stringer | 1977 | 36 | F | * | Lung, scalp, chest, bone, heart | No | No | Yes chemo and RT; 4750 Rad | 11 years | Death, pulmonary metastasis |
| 13 | Fischer | 1982 | 66 | F | 2 | No | No | No | No | 4 years | Alive, no evidence of disease |
| 14 | Berlin | 1984 | 60 | M | 3 | Lung, liver | No | No | No | 1 month | Death, pulmonary embolism |
| 15 | Humphrey | 1987 | 45 | M | 2.5 | No | No | Yes, 6800 Rad | No | 3 years | Alive, no evidence of disease |
| 16 | Welk | 1991 | 35 | F | 5 | No | No | Yes, 64 Gy | No | 11 months | Alive, no evidence of disease |
| 17 | Song | 1991 | 54 | F | | No | No | * | * | * | Alive, no evidence of disease |
| 18 | Dzsinich | 1992 | 70 | F | * | No | No | * | * | 17 years | Alive, no evidence of disease |
| 19 | Dzsinich | 1992 | 54 | F | * | Lung | No | * | * | 9 months | Death, pulmonary metastasis |
| 20 | Saglik | 1992 | 61 | F | 6 | Lung | Yes (twice) | No | Yes, chemo | 5,5 years | Death, pulmonary metastasis |
| 21 | Stellard | 1992 | 64 | F | 7 | No | No | No | No | * | Alive, no evidence of disease |
| 22 | Bryard | 1993 | 2 | F | 2.5 | No | Yes (twice) | No | Yes, chemo | 12 years | Alive, no evidence of disease |
| 23 | Stambuk | 1993 | 48 | M | 4.1 | No | No | Yes, 50 Gy | No | 1 year | Alive, no evidence of disease |
| 24 | Reix | 1998 | 64 | M | 5 | Skin, lung, brain | No | Refused RT | Yes, chemo | 6 years | Death, pulmonary metastasis |
| 25 | Van Marle | 2004 | 85 | F | 3.6 | No | No | Refused RT | No | 3 months | Alive, no evidence of disease |

*No data available.

theirs included. Since then another nine have been published,[1,3,6–11] making this case the 25th case in 85 years since Van Lee[12] described the first case of leiomyosarcoma originating from the greater saphenous vein in 1919. These 25 cases are presented in Table 1. The median age was 54 years (range 2–85 years). Most cases occur in the fourth to sixth decade.

In previous reports describing leiomyosarcomas of the venous system in the lower extremities[1,5] no gender predisposition was observed, which was somewhat surprising taking into account that a 4:1 female to male ratio is observed when the leimyosarcoma is located in the inferior caval vein. With 25 cases reviewed we observe a 3:2 female to male ratio.

The median size of the tumours was 4.1 cm, with a range from 2 to 12 cm. This is much smaller compared with leiomyosarcoma of the inferior caval vein, with a median size greater than 10 cm.[3]

Metastases occurred in seven of the 25 cases, with the lungs being the predominant site, and were the main cause of death. Because leiomyosarcomas are slow-growing tumours they often stay undetected for a long time, which is probably one of the causes of the large proportion of patients with metastases.

If any form of adjuvant therapy is given it is usually radiotherapy. The level of evidence to support this is low because of the rarity of the disease. However Mutter *et al.*[13] state that radiotherapy, 55–70 Gy, should follow excision of primary leiomyosarcoma if it occurs in the femoral vein. We offered it to our patient but she refused to undergo radiotherapy. Chemotherapy is mostly reserved to treat metastasis, with a wide variety of regimes being used, but doxorubicin and methotrexate are most commonly used. Initially the metastases respond well to the therapy, but ultimately the patient dies.[1,9]

When reviewing the literature, survival is found to be limited, with median survival ranging from 2.5 to about 4 years.[1,3,5] With 25 cases reviewed we find an average survival of 4 years, which could be much better if corrected for the short follow-up periods in some reports. The longest disease-free survival reported is 17 years.[5]

In conclusion, it can be stated that leiomyosarcoma of the saphenous vein is a very rare tumour. Because of its location in a vein, and because of its slow-growing tendency, and therefore its high rate of metastasis, survival is limited. If metastases occur, the lungs are the predominant site. In terms of diagnosis, MRI and Doppler echography seem to be the imaging techniques of choice. Because of the rarity of the disease there can be no conclusions drawn with regard to the necessity of adjuvant therapy. Radiotherapy is, however, frequently offered and seems beneficial.

Wide local excision with selective venous reconstruction, and adjuvant local radiotherapy, probably offers the only hope for prolonged survival.

### References

1. Reix T, Sevestri-Pietri MA, Szychta P, Pietri J. Primary malignant tumors of the venous system in the lower extremities. *Ann Vasc Surg* 1998; 12(6): 589–96.
2. Louail B, Vautier-Rodary R, *et al*. Value of imaging in early diagnosis of peripheral vein tumors. *J Radiol* 1998; 79(11): 1387–91.
3. Dzsinich C, Gloviczki P, van Heerder JA, *et al.* Primary venous leiomyosarcoma: a rare but lethal disease. *J Vasc Surg* 1992; 15(4): 595–603.
4. Perl L. Ein fall von sarkom der vena cava inferior. *Virchow's Arch* [A] 1871; 53: 378–83.
5. Humphrey M, Neff J, Lin F, Krishan L. Leiomyosarcoma of the saphenous vein. A case report and review of the literature. *J Bone Joint Surg Am* 1987; 69(2): 282–6.
6. Stambuk J, Oddo D, Perez R, Bravo M. Leiomyosarcoma of the saphenous vein. *Rev Med Chil* 1993; 121(6): 673–6.
7. Byard RW, Bourne AJ, Philips GE, Rice MS, Davey RB. Leiomyosarcoma of the saphenous veinin a child with 12-year follow-up. *J Pediatr Surg* 1993; 28(2): 271–4.
8. Stallard D, Sundararam M, Johnson FE, Janney C. Case report 747: leiomyosarcoma of great saphenous vein. *Skeletal Radiol* 1992; 21(6): 399–401.
9. Saglik Y, Icli F, Uluoglu O, Isiklar ZU. Leiomyosarcoma of the great saphenous vein. *Int Orthop* 1992; 16(2): 185–7.
10. Song KY, Jang YW, Kim MK, Lee GY, Sung RH. Leiomyosarcoma arising in the great saphenous vein: a case report. *J Kor Med Sci* 1991; 6(4): 372–5.
11. Welk E, Bindewald H, Berndt R. Vascular leiomyosarcoma: a surgical rarity. *Chirurgie* 1991; 62(3): 223–5.
12. Van Ree A. Phlebosarcoma racemosum. *Ned Tijdschr Geneeskde* 1919; 63: 759–67.
13. Mutter D, Limacher JM, Beaujeux R, *et al.* Primary leiomyosarcoma of the femoral vein. Therapeutic aspects. *J Chir (Paris)* 1994; 131(11): 457–60.
14. Berlin O, Stener B, Kindblom LG, Angervall L. Leiomyosarcomas of venous origin in the extremities. A corrolated clinical, roentgenologic, and morphologic study with diagnostic and surgical implications. *Cancer* 1984; 54(10): 2147–59.
15. Fischer MG, Gelb AM, Nussbaum M, Haveson S, Ghali V. Primary smooth muscle tumors of venous origin. *Ann Surg* 1982; 196(6): 720–4.
16. Stringer BD. Leiomyosarcoma of artery and vein. *Am J Surg* 1977; 134(1): 90–4.
17. Gross E, Horton MA. Leiomyosarcoma of the saphenous vein. *J Pathol* 1975; 116(1): 37–41.
18. Jernstrom P, Gowdy RA. Leiomyosarcoma of the long saphenous vein. *Am J Clin Pathol* 1975; 63(1): 25–31.
19. Hughes S. A case of leiomyosarcoma of the long saphenous vein. *Vasc Surg* 1973; 7(2): 71–3.
20. Szasz IJ, Barr R, Scobie TK. Leiomyosarcoma arising from veins:two cases and a review of the literature on venous neoplasms. *Can J Surg* 1969; 12(4): 415–9.
21. Leu HJ, Nipkow P. Malignant primary vein tumors. *Angiologica* 1969; 6(5): 302–9.

22. Allison MF. Leiomyosarcoma of the femoral vein. Report of a case in a child. *Clin Pediatr (Philadelphia)* 1965; 66: 28–31.
23. Christiansen J. Malignant tumors originating from the venous system. Review and a case of leiomyosarcoma from the long saphenous vein. *Ugeskr Laeger* 1964; 126: 483–5.
24. Dorfman HD, Fishel ER. Leiomyosarcoma of the greater saphenous vein. Report of a case and review of the literature. *Am J Clin Pathol* 1963; 39: 73–8.
25. Smout MS, Fisher JH. Leiomyosarcoma of the saphenous vein. *Can Med Assoc J* 1960; 83: 1066–7.

## References

[PDF_00311] ALLISON M. F. (1965). LEIOMYOSARCOMA OF THE FEMORAL VEIN. REPORT OF A CASE IN A CHILD.. Clin Pediatr (Phila).

[PDF_00287] Berlin O., Stener B., Kindblom L. G., Angervall L. (1984). Leiomyosarcomas of venous origin in the extremities. A correlated clinical, roentgenologic, and morphologic study with diagnostic and surgical implications.. Cancer.

[PDF_00266] Byard R. W., Bourne A. J., Phillips G. E., Rice M. S., Davey R. B. (1993). Leiomyosarcoma of the saphenous vein in a child with 12-year follow-up.. J Pediatr Surg.

[PDF_00314] CHRISTIANSEN J. (1964). MALIGNE TUMORER UDGAET FRA VENESYSTEMET. EN OVERSIGT OG ET TILFAELDE AF LEIOMYOSARKOM UDGAET FRA VENA SAPHENA MAGNA.. Ugeskr Laeger.

[PDF_00254] Dzsinich C., Gloviczki P., van Heerden J. A., Nagorney D. M., Pairolero P. C., Johnson C. M., Hallett J. W., Bower T. C., Cherry K. J. (1992). Primary venous leiomyosarcoma: a rare but lethal disease.. J Vasc Surg.

[PDF_00292] Fischer M. G., Gelb A. M., Nussbaum M., Haveson S., Ghali V. (1982). Primary smooth muscle tumors of venous origin.. Ann Surg.

[PDF_00297] Gross E., Horton M. A. (1975). Leiomyosarcoma of the saphenous vein.. J Pathol.

[PDF_00302] Hughes S. (1973). A case of leiomyosarcoma of the long saphenous vein.. Vasc Surg.

[PDF_00259] Humphrey M., Neff J., Lin F., Krishnan L. (1987). Leiomyosarcoma of the saphenous vein. A case report and review of the literature.. J Bone Joint Surg Am.

[PDF_00299] Jernstrom P., Gowdy R. A. (1975). Leiomyosarcoma of the long saphenous vein.. Am J Clin Pathol.

[PDF_00308] Leu H. J., Nipkow P. (1969). Malignant primary vein tumors.. Angiologica.

[PDF_00251] Louail B., Vautier-Rodary R., Gondry-Jouet C., Westeel A., Filloux-Morfaux V., Auquier M., Audebert M., Reix T., Pietri J., Remond A. (1998). Apport de l'imagerie dans le diagnostic précoce des tumeurs veineuses périphériques.. J Radiol.

[PDF_00284] Mutter D., Limacher J. M., Beaujeux R., Meyer A., Roy C., Bergerat J. P., Marescaux J. (1994). Léiomyosarcome primitif de la veine fémorale--aspects thérapeutiques.. J Chir (Paris).

[PDF_00247] Reix T., Sevestre H., Sevestri-Pietri M. A., Szychta P., Pietri J. (1998). Primary malignant tumors of the venous system in the lower extremities.. Ann Vasc Surg.

[PDF_00273] Saglik Y., Icli F., Uluoglu O., Isiklar Z. U. (1992). Leiomyosarcoma of the great saphenous vein.. Int Orthop.

[PDF_00322] Smout M. S., Fisher J. H. (1960). Leiomyosarcoma of the Saphenous Vein.. Can Med Assoc J.

[PDF_00276] Song K. Y., Jang Y. W., Kim M. K., Lee G. Y., Sung R. H. (1991). Leiomyosarcoma arising in the great saphenous vein--a case report.. J Korean Med Sci.

[PDF_00270] Stallard D., Sundaram M., Johnson F. E., Janney C. (1992). Case report 747: Leiomyosarcoma of great saphenous vein.. Skeletal Radiol.

[PDF_00263] Stambuk J., Oddó D., Pérez R., Bravo M. (1993). Leiomiosarcoma de vena safena.. Rev Med Chil.

[PDF_00295] Stringer B. D. (1977). Leiomyosarcoma of artery and vein.. Am J Surg.

[PDF_00304] Szasz I. J., Barr R., Scobie T. K. (1969). Leiomyosarcoma arising from veins:two cases and a review of the literature on venous neoplasms.. Can J Surg.

[PDF_00279] Welk E., Bindewald H., Berndt R. (1991). Das vasculäre Leiomyosarkom--eine chirurgische Rarität.. Chirurg.

